# Fibrolase: Trials and Tribulations

**DOI:** 10.3390/toxins2040793

**Published:** 2010-04-20

**Authors:** Francis S. Markland, Steve Swenson

**Affiliations:** 1 Department of Biochemistry and Molecular Biology, Cancer Research Laboratory, Keck School of Medicine, University of Southern California, 1303 N. Mission Rd., Los Angeles, CA 90033, USA; 2 USC/Norris Comprehensive Cancer Center, Keck School of Medicine, University of Southern California, Los Angeles, CA 90033, USA; Email: sswenson@usc.edu

**Keywords:** fibrolase, alfimeprase, thrombolysis, peripheral arterial occlusion, animal models, central venous access device occlusion, stroke, alpha2 macroglobulin, metalloproteinase

## Abstract

Fibrolase is the fibrinolytic enzyme isolated from *Agkistrodon contortrix contortrix* (southern copperhead snake) venom. The enzyme was purified by a three-step HPLC procedure and was shown to be homogeneous by standard criteria including reverse phase HPLC, molecular sieve chromatography and SDS-PAGE. The purified enzyme is a zinc metalloproteinase containing one mole of zinc. It is composed of 203 amino acids with a blocked amino-terminus due to cyclization of the terminal Gln residue. Fibrolase shares a significant degree of homology with enzymes of the reprolysin sub-family of metalloproteinases including an active site homology of close to 100%; it is rapidly inhibited by chelating agents such as EDTA, and by alpha2-macroglobulin (α2Μ). The enzyme is a direct-acting thrombolytic agent and does not rely on plasminogen for clot dissolution. Fibrolase rapidly cleaves the A(α)-chain of fibrinogen and the B(β)-chain at a slower rate; it has no activity on the γ-chain. The enzyme exhibits the same specificity with fibrin, cleaving the α-chain more rapidly than the β-chain. Fibrolase was shown to have very effective thrombolytic activity in a reoccluding carotid arterial thrombosis model in the canine. A recombinant version of the enzyme was made in yeast by Amgen, Inc. (Thousand Oaks, CA, USA) and called alfimeprase. Alfimeprase is identical to fibrolase except for a two amino acid truncation at the amino-terminus and the insertion of a new amino-terminal amino acid in the truncated protein; these changes lead to a more stable enzyme for prolonged storage. Alfimeprase was taken into clinical trials by Nuvelo, Inc. (San Carlos, CA), which licensed the enzyme from Amgen. Alfimeprase was successful in Phase I and II clinical trials for peripheral arterial occlusion (PAO) and central venous access device (CVAD) occlusion. However, in Phase III trials alfimeprase did not meet the expected end points in either PAO or CVAD occlusion and in a Phaase II stroke trial, and Nuvelo dropped further development in 2008.

## 1. Introduction and Background

Kornalik in Czechoslovakia in 1966 was the first to report fibrinolytic activity in *Agkistrodon contortrix contortrix* (southern copperhead snake) venom [1], although earlier Didisheim and Lewis had suggested that snake venoms may contain fibrinolytic activity that should be useful for clinical application since it may not be inactivated by inhibitors in mammalian blood [2]. However, it was not until much later that the fibrinolytic enzyme was purified. The Markland laboratory, at the University of Southern California (USC), first identified the enzyme in 1982 [3] and subsequently purified it [4]. Fibrolase was the name given to this fibrinolytic metalloproteinase. 

Fibrolase is a direct acting, 23-kDa fibrinolytic enzyme that cleaves the Aα-chain of fibrinogen (primary cleavage site Lys-Leu bond at residues 413–414). The enzyme also cleaves the Bβ-chain at a slower rate, but it has no effect on the γ-chain [5]. The enzyme was purified by 3-step HPLC method involving hydrophobic interaction HPLC, hydroxyapatite HPLC and cation exchange HPLC [4,6]. Fibrolase is a member of family M12, subfamily B (the reprolysins), a grouping of proteolytic enzymes composed of many enzymes of snake venom origin. The active site of the molecule has been identified in the zinc-binding region of fibrolase, spanning amino acids 141–170 [7,8].

Fibrolase differs from the plasminogen activator-based thrombolytics since it acts directly on fibrin; it does not rely on activation of plasminogen (fibrolase neither activates nor degrades plasminogen) [5]. Fibrolase is a metalloproteinase and not a serine protease [5]. Therefore, it is not inhibited by the blood serine protease inhibitors, SERPINS [9], which are targeted to the blood clotting and fibrinolytic serine proteinases. However, incubation of fibrolase with plasma leads to inhibition of fibrinolytic activity due to the covalent binding of fibrolase by alpha-2 macroglobulin (α2M) [10,11]. The amino acid sequence of fibrolase was determined in a collaboration between the Markland laboratory and investigators at Chiron Corp. (Emeryville, CA, USA) [7], and the sequence clearly shows that the enzyme is a member of the M12 group of metalloproteinases, the reprolysins. Further, the sequence reveals that there is a complete absence of consensus sites for N-linked glycosylation (Asn-X-Ser/Thr). Separate studies indicated that the enzyme has no thrombin-like activity, no protein C activation activity, no activation nor degradation of plasminogen, no platelet aggregating activity *in vitro*, no hemolytic activity, and importantly no hemorrhagic activity [12].

## 2. Evolution of Fibrolase as a Thrombolytic Agent

Based on the direct action of fibrolase on fibrin and the lack of inhibition by blood SERPINS, it appeared that fibrolase should be an effective thrombolytic agent. With this as a background, Dr. Markland and Dr. Benedict Lucchesi, a well-known authority on the use of animal models for evaluation of thrombolytic and anti-platelet agents at the University of Michigan, agreed to collaborate on a study to assess the potential thrombolytic activity of fibrolase in a canine reoccluding carotid arterial thrombosis model. Dr. Markland took fibrolase to the University of Michigan and with Dr. Lucchesi demonstrated that the enzyme isolated from snake venom had excellent lytic activity in this 100% reoccluding arterial thrombosis model [13]. The collaborative study showed that the enzyme rapidly lysed clots in the carotid artery when administered at a dose of 4 mg/kg in a volume of 3 mL infused over a 5 minute period proximal to the site of the thrombus. In this model system, arteries infused with fibrolase in five of five dogs were shown to be cleared of the occluding thrombus within 6 minutes of initiation of lytic agent infusion. In the contralateral carotid artery that received only vehicle, the vessels remained occluded throughout the course of the experiment. By comparison, animals receiving anisoylated plasminogen streptokinase activator complex (APSAC) lysed the thrombus within 26 minutes of infusion. Five minutes after completing fibrolase administration and in one group of the two groups administered APSAC, a fibrinogen receptor antagonist, 7E3 (0.8 mg/kg) was administered intravenously to inhibit platelet aggregation and prevent reocclusion of the thrombolytic-treated arteries. After administration of 7E3, four of five carotid arteries in the dogs treated with fibrolase remained open for the remainder of the experiment, and six of the six arteries treated with APSAC. The average time to thrombus resolution for APSAC was 26 minutes while for recombinant fibrolase it was 6 minutes. It was concluded from these studies that fibrolase is an active lytic agent and rapidly lyses carotid arterial thrombi without evidence of hemorrhage or compromise of the hemodynamic system. In summary, we concluded that in combination with antiplatelet therapy, fibrolase offers a unique mechanism for clot dissolution that may provide an alternative to plasminogen activator-based thrombolysis and could have significant potential for clinical application. A sideline to this study was a problem that was experienced with the recombinant version of the enzyme produced by Chiron under a subcontract from Marion Laboratories, Kansas City, MO (later known as Marion Merrell Dow). To exploit the commercial potential of fibrolase, Marion Laboratories supported research by the Markland lab and subcontracted Chiron to produce a recombinant version of fibrolase. After trying several different expression methods, Chiron settled on a yeast expression system and provided the recombinant version of the protein to Dr. Lucchesi. Unfortunately, the two dogs that were treated with the recombinant enzyme died shortly after treatment, leading to the suspicion that the recombinant protein contained an impurity. Based on these findings Dr. Markland contacted Dr. Pablo Valenzuela at Chiron, who was Vice President of Research at that time, and obtained more of the recombinant protein that was passed through a detox column to remove any potential endotoxin contamination. Dr. Markland then took the purified recombinant enzyme back to the University of Michigan to examine its activity. This time the enzyme worked, suggesting that the original batch of recombinant fibrolase had been contaminated. The results of this study were published jointly with Dr. Lucchesi’s laboratory in 1994 [13]. But, the damage had already been done. On the basis of the failed test in the two dogs and other Company related issues, Marion Laboratories decided to pull out of the project. Funding for the project was halted and the research project lay fallow for several years. However, Dr. Chris Toombs, Research Scientist at Amgen, read the paper describing the thrombolytic activity of recombinant fibrolase [13] and contacted Dr. Markland about possible interest by Amgen in the clinical potential of the enzyme. A Material Transfer Agreement and a Research Agreement between Amgen and the University of Southern California (USC) was put into place to develop the clinical potential of fibrolase; this agreement was in place from 1996 until 2001. Table 1 summarizes timelines for the evolution of events involved in the long and winding road to clinical trials.

**Table 1:** Chronology of fibrolase-alfimeprase evolution to clinical trials.

1982	Fibrolase first identified in the Markland laboratory using molecular sieve chromatography of Southern copperhead venom
1986	US Patent No. 4,610,879 issued to F.S. Markland, Jr. and N.K. Reddy: Fibrinolytic Enzyme from snake venom
1986	Marion Laboratories, Inc., Kansas City, MO (Marion Merrell Dow, MMD) signs a subcontract with Chiron to produce r-fibrolase in yeast or bacteria
1996–2001	Research agreement between USC and Amgen. Amgen produces an altered recombinant form of fibrolase in yeast and renames it alfimeprase
2002	Nuvelo (then know as Hyseq) obtains rights to alfimeprase from Amgen and initiates clinical trials (20 years after protein is discovered). US FDA grants orphan drug status to alfimeprase for PAO
2004	Amgen licenses alfimeprase to Nuvello with future milestone payments due to Amgen (November 2004)
2006	FDA grants Nuvelo fast track designation for alfimeprase for the treatment of PAO in NAPA-3 Phase III trial (January 2006)
2006	Bayer Healthcare signs $385M deal for worldwide (non-USA) rights to alfimeprase with Nuvelo, $50M upfront based on Phase II PAO results (January 2006)
2007	Bayer pulls out of agreement with Nuvello; Nuvello indicates Phase II study in acute ischemic stroke will commence at end of year (June 2007)
2008	Nuvelo abandons development of alfimeprase after it fails to meet endpoints in Phase III PAO and CO trials and low enrollment in the Phase II stroke treatment trial (March 2008)

## 3. The Fibrolase—Alfimeprase Connection

At Amgen Dr. Toombs led a group of scientists that produced a recombinant version of fibrolase truncated by two amino acids at the amino-terminus that was ultimately called alfimeprase; an interim terminology of NAT (natural acting thrombolytic) was also used. The truncation was necessitated by the presence of several isoforms of the enzyme encountered by Amgen, and previously experienced by the Markland laboratory with the natural enzyme [14,15]. Using an *E. coli* expression system, Amgen observed the isoforms during isolation of fibrolase. Additionally, in the *E. coli* system a significant percentage of fibrolase was retained in inclusion bodies resulting in a very low yield of active enzyme. In view of these difficulties, a eukaryotic host (a yeast system) was examined for its ability to produce active alfimeprase. The yeast *Pichia pastoris* was chosen as it has been widely used and served as an effective host for heterologous expression of recombinant proteins [8]. In the *P. pastoris* expression system, the synthetic gene for alfimeprase is incorporated into the genomic DNA of an untransformed yeast strain. A plasmid is used, which encodes alfimeprase DNA and the enzyme is expressed under the control of the alcohol oxidase 1 promoter, which is induced by the presence of methanol and is tightly regulated. Methanol not only serves as the sole carbon source for this strain of yeast but also induces expression of the target protein. The yeast cells are cultured in an induction medium that contains methanol. The secreted alfimeprase is purified from the media and formulated into buffer containing Zn^2+^ at physiologic pH.

Alfimeprase contains 201 amino acids with an N-terminal sequence of SFPQR- as compared to fibrolase, which contains 203 amino acids with an N-terminal sequence that begins with EQRFPQR-, otherwise alfimeprase is identical in structure and enzymatic activity to fibrolase. The CAS Registry Service has assigned registery number 259074-76-5 with the index name 3-203 fibrolase [3-serine] (*Agkistrodon contortrix contortrix*, recombinant). X-ray crystallographic data and modeling of the structure of fibrolase indicated that the amino-terminus of fibrolase was free to move about in three-dimensional space presumably causing instability of the protein [16]. A more stable protein was produced as a result of the two amino acid truncation and substitution of Ser for Arg at the new amino-terminus. This led to stability during long-term storage as well as eliminating the isoform problem, which originated because of sequence variations at the amino-terminus [15]. 

**Figure 1:**
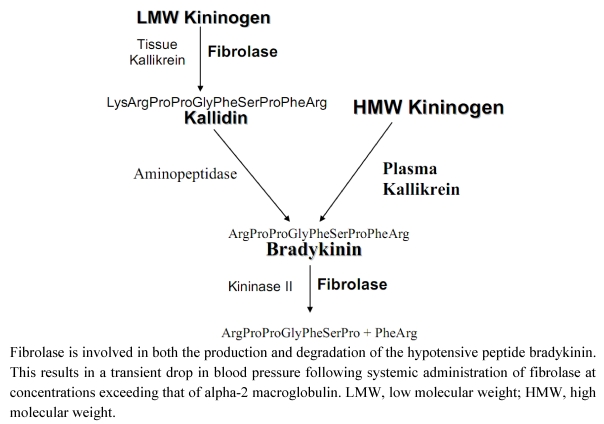
Effect of fibrolase on the bradykinin metabolism pathway.

Dr. Toombs, at Amgen, noted that during studies on the lytic activity of fibrolase in a guinea pig model, there was transient hypotension following intra-arterial administration of the enzyme. However, this could be prevented by treatment with a bradykinin antagonist. To identify the possible mechanism involved in fibrolase-mediated hypotension, the catalytic activity of fibrolase against proteins in the bradykinin synthetic pathway were assessed. There are two biochemical routes that lead to the synthesis of bradykinin (Figure 1) [17]. Plasma kallikrein directly cleaves high molecular weight kininogen (HMWK) generating bradykinin. However, tissue kallikrein can cleave low molecular weight kininogen (LMWK) to form kallidin a decapeptide. The amino-terminal lysine of kallidin is then removed by plasma aminopeptidase to form bradykinin. Bradykinin is subsequently cleaved to the inactive heptapeptide Arg-Pro-Pro-Gly-Phe-Ser-Pro by kininase II. The Markland lab determined that fibrolase promotes bradykinin formation by cleavage of LMWK with the formation of kallidin. Kallidin is a relatively poor substrate for fibrolase. Fibrolase also cleaves bradykinin to the inactive heptapeptide Arg-Pro-Pro-Gly-Phe-Ser-Pro. Thus, the transient nature of the production and subsequent degradation of bradykinin mimics findings observed with blood pressure alterations in animals treated with fibrolase. Fibrolase appears to promote bradykinin generation through the LMWK pathway (Figure 1), but has no effect on HMWK. Although kallidin is a poor substrate for fibrolase, fibrolase can cleave bradykinin effectively. Analysis of the bradykinin cleavage products by mass spectrometry confirmed that fibrolase cleaves between residues Pro7-Phe8 to form the inactive heptapeptide (shown above). In summary, fibrolase can generate kallidin from LMW kininogen and after bradykinin is generated by aminopeptidase action, fibrolase cleaves and inactivates bradykinin in a manner similar to kininase II. This results in the transient hypotension observed following fibrolase treatment.

## 4. Alfimeprase *in Vitro* Studies

Dr. Toombs led the preclinical studies of alfimeprase at Amgen. He initiated *in vivo* studies to characterize thrombolytic activity of the enzyme in a number of different animal models and he carried out *in vitro* studies to characterize the inhibitory activity of α2M on alfimeprase. Dr. Toombs showed that in the presence of 3-fold molar excess α2M, alfimeprase rapidly forms a complex with the inhibitor. Using SDS-PAGE and Western blotting with an antibody to alfimeprase, he demonstrated that complex formation begins within 5 s and is complete by 1.5 min [18]. The interaction between α2M and alfimeprase involves covalent bond formation with 1:1 stoichiometry between enzyme and inhibitor, identical to the interaction of α2M with fibrolase. The capacity of serum to bind and neutralize alfimeprase was determined based on the concentration of α2M in human serum (~100 to 300 mg/dL). The binding capacity of alfimeprase for α2M in human serum was determined and was initially estimated to be 40–50 μg alfimeprase per mL of human serum [8]. In a study carried out at Amgen, the effect of alfimeprase on human plasma fibrinogen was examined. Plasma was collected from 20 human donors and fibrinogen quantified in all samples prior to the addition of alfimeprase; following addition of alfimeprase fibrinogen levels in the plasma samples were measured at 30, 60, and 120 minutes. Plasma fibrinogen was not affected by incubation with alfimeprase at 10 μg/mL (alfimeprase was completely inhibited by α2M). However, with alfimeprase at 100 μg/mL incubation of plasma resulted in complete degradation of fibrinogen in all plasma samples within 30 minutes. This suggested that at 100 μg/mL alfimeprase, the capacity for α2M to bind and inactivate the enzyme had been exceeded. These studies point out the importance of not over-titrating α2M by alfimeprase since the presence of an active enzyme in blood could potentially cause serious side effects during clinical trials.

## 5. Alfimeprase Animal Model Studies

A number of animal model studies were conducted at Amgen: alfimeprase was investigated in a rat acute carotid artery thrombosis model, two piglet acute carotid artery thrombosis models, a dog acute/subacute carotid arterial thrombosis model, and a baboon model involving acute thrombosis in exteriorized arteriovenous shunts [8]. Alfimeprase was delivered locally in all of these models to avoid the inhibitory effect of α2M, which would have inactivated the enzyme following intravenous administration (see description in Table 2) [8]. 

**Table 2:** Key Findings from Selected Animal/Human Pharmacology Studies ^1,2^.

Species	Model	Design	Key Findings
Human	*In vitro* clot lysis	Preformed human whole blood clots in tubes or mounted in PTFE graft	Rate of clot lysis is directly proportional to the quantity of alfimeprase added or infused into the graft segment.
Rat	Acute carotid thrombosis	Alfimeprase (2 mg total) *vs.* UK (250 U/min)	71% incidence (10/14) of clot lysis in 6.3 minutes in alfimeprase group *vs.* 87% (13/15) in 33.5 minutes in UK group.
Piglet	Acute carotid thrombosis; thrombus aged for 30 minutes	Alfimeprase (5 mg total) *vs.* tPA (2 mg/kg)	100% incidence (11/11) of clot lysis in 4.4 minutes with alfimeprase *vs.* 70% incidence (7/10) in 17.8 minutes with tPA.Average blood loss: alfimeprase = 1.7 mL, t-PA = 17.1 mL
Piglet	Acute carotid thrombosis; thrombus aged for 30 minutes	Alfimeprase (3 and 6 mg total) *vs.* UK (500 U/min and 2,000 U/min)	83% (10/12) and 91% (10/11) incidence of clot lysis in 7.1 and 10.0 minutes for the 3 and 6 mg groups, respectively.; 17% (2/12) and 25% (3/12) incidence of clot lysis in 39.0 and 28.0 minutes in 500 U/min and 2000 U/min groups, respectively.
Dog	Acute/ subacute carotid thrombosis thrombus aged for 30 minutes or 24 hours	30 minute: Alfimeprase (2 mg/kg) *vs.* UK (4500 U)	In 30 minute old clots, alfimeprase resulted in 100% incidence (7/7) of clot lysis in 7.5 minutes *vs.* UK with 83% incidence (5/6) in 38.8 minutes.
		24 hour: Alfimeprase (3.8 mg/kg) *vs.* UK (13,500 U)	In 24-hour clots, 75% success (6/8) in 72 min with alfimeprase *vs.* 67% success in 120 minutes using UK.
Baboon	Acute thrombosis of Dacron ® grafts in exteriorized arteriovenous shunts	Alfimeprase (1 or 10 mg/mL) *vs.* UK (5,000 or 50,000 U/mL)	With 1 mg/mL alfimeprase, flow restored in average of 15.5 minutes and with 10 mg/mL, flow restored in 8.7 minutes. With 5000 U/mL UK, flow restored in average of 28.3 minutes and with 50,000 U/mL, flow restored in 11.7 minutes. A decrease (14% to 17%) in fibrinogen and an increase (79% to 176%) in D-dimer was noted in the animals treated with UK, whereas negligible changes in these parameters (±10% of baseline) occurred in alfimeprase treated animals.
		Agents are infused into graft segment at 1 μL/min until flow is restored	

^1^ Abbreviations: PTFE = polytetrafluoroethylene (Teflon); UK = urokinase; tPA = tissue plasminogen activator; ^2^ Taken from [8], with permission from Bentham Science Publishers.

Amgen investigators also examined a porcine model of peripheral arterial occlusion (PAO) in which the carotid artery of adult pigs (~20 cm long) is thrombosed by balloon injury plus thrombin administration and stasis. The animals are allowed to recover for 4 days to form a stabilized thrombus. In this model of peripheral arterial occlusion (PAO) both the size and age of the thrombus are close approximations of the size and duration of ischemic symptoms reported in the largest clinical trial of thrombolysis in PAO, the Thrombolysis Or Peripheral Arterial Surgery (TOPAS) study [19]. In the porcine model a multiple side-hole catheter is advanced for drug delivery into the thrombus. With a dose of 30 mg alfimeprase there is close to complete thrombus resolution and flow restoration within 30 minutes. By comparison, UK 250,000 U bolus plus 4,000 U/min infusion did not give comparable results despite continuing infusion for up to 4 hours. 

Separately, a study was carried out by Dr. Lucchesi and colleagues to compare the effect of alfimeprase and rt-PA on extent of myocardial reperfusion injury [20]. This study was based on the hypothesis that thrombolysis in the absence of plasmin generation could result in improved myocardial salvage. The authors examined the thrombolytic effect of recombinant t-PA (rt-PA) (0.022 mg/kg, 10% delivered as a loading dose and 90% infused over 60 minutes by intracoronary administration) *vs.* alfimeprase (0.5 mg/kg over 1 minute by intracoronary administration) in a canine model of electrolytic injury to the left circumflex coronary artery (LCX). Both agents induced thrombolysis, with alfimeprase being more rapid with onset of reperfusion in 1.5 ± 0.6 *vs.* 10.1 ± 2.1 minutes for rt-PA. In the absence of adjuvant anti-platelet therapy, however, time to reocclusion was much shorter with alfimeprase (3.2 ± 0.5 minutes *vs.* 77.5 ± 31.9 minutes for rt-PA). In a separate group of animals, the presence of a glycoprotein IIb/IIIa platelet receptor antagonist dramatically prolonged time to reocclusion for both agents: alfimeprase was 163.3 ± 27.4 min and rt-PA was 142.8 ± 34.5 min. The effect on myocardial infarct size was examined after dogs were exposed to 60 min of LCX occlusion followed by intracoronary agent delivery, and after 4 h of reperfusion, infarct size was measured. The myocardial infarct size after alfimeprase was 19.9% ± 3.6% of risk region and 32.2% ± 4.0% after rt-PA, *vs.* 18.5% ± 3.3% for saline control [20]. Thus, local administration of alfimeprase produced a significant advantage both in the shorter time to onset of reperfusion as well as smaller size of myocardial infarct as compared to rt-PA. The generation of plasmin by rt-PA could be associated with secondary proteolytic effects that lead to larger infarct size. The alfimeprase results were obtained with no evidence of altered hemostasis or bleeding at remote sites since α2M limits alfimeprase lytic action to the site of application. Others have suggested that plasminogen activators activate enzyme systems with pro-inflammatory effects, which contribute to the pathogenesis of ischemia-reperfusion injury. Thus, rt-PA appears to be associated with greater risk of myocardial reperfusion injury than alfimeprase [20].

An acute ischemia-reperfusion stroke model in rats was performed to evaluate the activity of alfimeprase *vs.* rt-PA following reperfusion of the middle cerebral artery after 5-hour occlusion [21]. The blinded study compared 10 minute infusion immediately after reperfusion using alfimeprase at 0.03, 0.1 and 0.3 mg/kg *vs.* rt-PA at 1 mg/kg. Analysis of brain sections of treated animals with an alfimeprase dose of 0.03 mg/kg revealed that the hemorrhagic transformation frequency, neurological deficit and mortality rate were much lower than for rt-PA; at higher doses of alfimeprase there were no differences *vs.* rt-PA. There were no significant differences in infarction and blood-brain barrier permeability when comparing control, 0.1 mg/kg alfimeprase and 1 mg/kg rt-PA. This study indicated a similar safety profile of alfimeprase and rt-PA [21]. 

In conclusion, the *in vivo* studies revealed that alfimeprase could be used to successfully lyse clots in a number of animal models of arterial thrombosis (Table 2). In all models alfimeprase was administered locally to avoid α2M inhibition and the enzyme was shown to act across species. Alfimeprase did not increase hemorrhage in a rat ischemic stroke model *vs.* recombinant t-PA (rt-PA), nor lead to other neurological problems [21]. Thrombi were effectively and quickly cleared, and alfimeprase did not promote rethrombosis. Since alfimeprase is rapidly inactivated in the general circulation by α2M, the enzyme appears to offer promise as a safe, effective and specific agent for thrombolysis when administered locally at the site of the thrombus [22]. A number of US patents have been issued for alfimeprase [23].

## 6. Clinical Trials

The *in vitro* and animal model studies on fibrolase and alfimeprase were aimed to advance the protein toward clinical trials. Nuvelo (at that time known as Hyseq; San Carlos, CA), obtained in 2002 the rights to alfimeprase from Amgen and initiated clinical trials. Twenty years had elapsed from the time the protein was first reported by the Markland laboratory [3] and the start of clinical trials (Table 1). A Phase I clinical trial was designed to evaluate the safety profile, pharmacokinetics, and thrombolytic activity of alfimeprase in patients with chronic peripheral arterial occlusion (PAO). PAO or “leg attack” is caused by a blood clot that causes blockage of arterial blood flow to a lower limb. This underlying peripheral arterial disease, if untreated can lead to nerve and muscle damage, gangrene and in severe cases amputation and death. Treatment involves rapid restoration of arterial patency and blood flow, as well as limb preservation. PAO affects more than 100,000 people per year in the United States and an equal number in Europe.

The Phase I trial was a multicenter, open-label, single-dose, dose-escalation study, involving 20 patients with worsening symptoms of lower extremity ischemia who were treated with alfimeprase in five escalating dose cohorts (0.025, 0.05, 0.1, 0.3, and 0.5 mg/kg) [18]. The United States FDA granted orphan drug status to alfimeprase for the Nuvelo clinical trial for PAO. Orphan drug status was created by the FDA for agents used to treat diseases that occur in less than 200,000 cases or where there is no hope of recovering the development costs for the Company, so there is little financial incentive to develop these drugs. Granting orphan drug status provides the manufacturer specific financial incentives to provide the drug. A pulsed infusion delivery modality for alfimeprase was used either intraarterial or sometimes intrathrombus. The primary endpoint was safety as assessed by adverse event rates. Additional safety assessments included α2M, and anti-alfimeprase antibodies for as long as 3 months after treatment. Pharmacokinetic parameters were evaluated with use of an assay that measures free and α2M-bound (total) alfimeprase. The study was conducted in 7 US hospitals by Nuvelo and was completed in March of 2003. There were no deaths and none of the patients experienced adverse hemorrhagic events. Further, the mean plasminogen and fibrinogen concentrations were not substantially altered by treatment. There were three transient, treatment-related adverse events; all were mild and in the same patient. The half-life for alfimeprase ranges from 11 to 54 minutes (median, 25 min) in patients with PAO. Serum α2M decreased transiently in a dose-dependent manner after treatment, but returned to normal ~14 days after treatment. Alfimeprase doses were selected to be within the alfimeprase-binding capacity of α2M, which was 1.71 mg/kg. The Phase I trial demonstrated that alfimeprase in doses as high as 0.5 mg/kg (within the binding capacity of α2M) was generally safe in patients with chronic PAO. There were no bleeding complications and no systemic thrombolysis was noted. Further, the stable fibrinogen concentrations suggest that alfimeprase action is limited to the target thrombus. There were no instances of anaphylaxis and no anti-alfimeprase antibodies three months after alfimeprase administration. None of the serious adverse events were attributed to alfimeprase. Thus, this Phase I clinical trial demonstrated that alfimeprase holds the potential to achieve dissolution of PAO with minimal risk of hemorrhage [24,25].

NAPA-1 (Novel Arterial Perfusion with Alfimeprase), which was a Phase II trial, commenced in July 2004, and was a multinational, open label, dose escalation trial in 113 acute PAO patients. The primary objective was safety, and a secondary objective was efficacy. Alfimeprase (0.1, 0.3 or 0.6 mg/kg, 2/3 then 1/3 of dose administered 2 h apart via side-hole catheter placed into the thrombus) was administered in 1 mL pulses at 1 pulse/min. Patients were enrolled within 14 days of symptoms and all patients were over 18 years old. Alfimeprase lysed thrombi at a rate of up to 76%, restored arterial flow up to 60% within 4 h of dosing and 52%–69% of patients avoided surgical intervention. There were no intracerebral hemorrhages or deaths, and only one major and three minor bleeds as far as Adverse Events (AE) that could possibly be attributed to alfimeprase (patients received aspirin and heparin). There was transient hypotension at high dose but this could be easily managed and α2M dropped 40%–60% but recovered by day 14 [25].

The Phase 3 Trials, NAPA-2 starting in April 2005, and NAPA-3 starting in April 2006, were overlapping, randomized, multinational trials, in which 0.3 mg/kg alfimeprase was compared to placebo. The intent was to enroll ~600 patients worldwide with symptom onset within 14 days. The primary endpoint was avoidance of open vascular surgery within 30 days of alfimeprase treatment; secondary endpoints were rate of arterial flow restoration at 4 h after drug administration and rate of improvement in ankle brachial index at 30 days (Table 3). The AE and severe AE (SAE) were major bleeding, intra-cranial hemorrhage, peripheral embolic events, all cause mortality and pharmacoeconomics such as length of hospital stay. NAPA-2, was a partial double-blind study with 4:3:1 randomization between intrathrombus alfimeprase, intrathrombus placebo, and perithrombus placebo. Alfimeprase was administered 2/3 of total dose given initially and then 1/3 dose given 2 h later. By comparison, NAPA-3 was a double-blind study with 1:1 randomization between intra-thrombus alfimeprase and intrathrombus placebo, using the same drug administration schedule, 2/3 of total dose and then 1/3 dose given 2 h later. In January 2006 alfimeprase was granted fast track designation for the NAPA-3 trial for PAO. Fast track designation facilitates development and expedited review of new drugs that demonstrate the potential to address an unmet medical need and are intended for treatment of serious or life-threatening conditions. During the NAPA-2 and -3 studies Dr. Fred Weaver, a vascular surgeon at USC and leader of the alfimeprase clinical trial at the Keck School of Medicine site at USC, and his colleagues, noticed that there was a correlation between the length of the thrombus and the success of lysis by alfimeprase: for short occlusion lengths (<10 cm or from 10 cm to <20 cm) the difference in flow restoration at 4 hours between alfimeprase and intrathrombus placebo was virtually nonexistent, whereas for an occlusion length of °40 cm the 4 hour flow restoration rate was 45.5% for alfimeprase and only 16.7% for placebo. This correlation also held when there was no early decline in circulating α2M level, where the 4-hour flow restoration rate for alfimeprase was 70%, whereas for placebo it was 37%. These results suggested to Weaver and colleagues that when alfimeprase is trapped in longer thrombi and is not released into the general circulation, where it interacts with α2M causing rapid depletion of α2M, the enzyme is degrading the thrombus leading to more rapid flow restoration in the longer thrombi [26]. These findings suggested that there may have been some flaws in the side hole catheter delivery mechanism and that alternative delivery methodologies with improved residence in the clot should be explored.

**Table 3:** NAPA-2 and NAPA-3 trials: primary and secondary endpoint results ^†^.

	NAPA-2			NAPA-3 (interim)	
	ALF* (n = 149)	**IT Placebo** (n = 113)	**PT Placebo** (n = 38)	**ALF** (n = 51)	**IT Placebo** (n = 51)
Rate of 30-day open vascular surgery avoidance	34.9%	37.2%	18.4%	29.4%	17.6%
Rate of restoration of arterial flow at four hours	46.3%	37.2%	15.8%	35.3%	23.5%
Rate of 30-day ABI** improvement	24.8%	23.0%	7.0%	11.8%	7.8%

^†^ Data adapted from Han, SM, Weaver, FA, *et al*. [26]; *ALF = alfimeprase, ABI = ankle-brachial index; ** The Ankle-Brachial Index is the ratio of blood pressure in the lower legs to the blood pressure in the arms. Lower blood pressure in the leg is an indication of blocked arteries (PAO).

In summary, the Phase III NAPA trials did not meet the primary endpoint and showed no significant difference between the intrathrombus alfimeprase and intrathrombus placebo groups [26]. The greater efficacy observed in longer clots and with a smaller drop in α2M levels, suggested that improved drug retention in the clot might improve lysis. AE profile in alfimeprase treated patients differed between NAPA-2 and NAPA-3 with the NAPA-3 trials being more favorable for alfimeprase. In both trials the majority of hypotensive episodes occurred within 15 minutes of alfimeprase administration without apparent clinical sequelae. The risk of hypotension increases with use of anti-hypertensives, particularly renin-angiotensin antagonists. Hypotension may be related to local bradykinin generation induced by alfimeprase as discussed above (see Figure 1). As far as hemorrhage, in NAPA-2 the majority were due to catheter site bleeding (23.0% intrathrombus alfimeprase *vs.* 10.8% intrathrombus placebo) and surgical bleeding (6.1% intrathrombus alfimeprase *vs.* 1.7% intrathrombus placebo). Major hemorrhage was higher with intrathrombus alfimeprase (5.4%) *vs.* intrathrombus placebo (0.9%). In NAPA-3 the catheter site bleeding was confirmed, but no surgical bleeding or major hemorrhage risk was observed. Finally, interim analysis of NAPA-3 showed favorable efficacy trends, but the sponsor decided the delivery method was not optimal and terminated the study. A further evaluation is warranted to improve and optimize the delivery system for alfimeprase to maximize retention of drug in the thrombus and increase lytic activity in PAO.

The second clinical application of alfimeprase was central venous access device (CVAD) occlusion. This is a big market as about five million catheters are positioned annually in the United States to deliver chemotherapy, nutrients, antibiotics and blood products, and up to 25% of them become occluded. Because of the direct fibrin degradation activity of alfimeprase, it was hypothesized that in patients with an occluded CVAD there would be rapid clot dissolution following alfimeprase treatment. A Phase II randomized, double-blind, active-control, multicenter, dose-ranging trial was initiated with patients enrolled between May 2003 and August 2004. Safety and efficacy of one or two instillations of 3 intraluminal doses of alfimeprase (0.3, 1.0 and 3.0 mg) or alteplase 2.0 mg were compared (alteplase is a version of recombinant t-PA developed by Genentech) [27,28]. The ability of alfimeprase to re-establish patency in 55 adult patients with occluded CVADs was investigated in this Phase II trial. Alfimeprase was shown to restore CVAD function in up to 60% of treated patients in less than 30 minutes, with the majority of these successes occurring in 15 minutes or less with 3 mg of alfimeprase. This rapid activity was associated with an acceptable and favorable safety profile. Alfimeprase treatment was well tolerated, with no intracranial hemorrhage or any other major hemorrhagic or embolic events reported for any of the patients. There were no adverse events that suggested a safety concern. In conclusion, this Phase II trial demonstrated that all three alfimeprase doses were more successful than alteplase at 5, 15 and 30 min during the first treatment. Alfimeprase at the 3.0 mg dose resulted in 40%, 50%, and 60% patency restoration rates at 5, 15, and 30 min, compared with 0%, 0%, and 23% for alteplase. Alfimeprase at 3.0 mg produced the highest patency rate at 120 min after the first (60%) and second (80%) doses (Table 4), and no major hemorrhagic or embolic events were reported [29,30]. 

**Table 4:** Clinical Potency of Alfimeprase *vs.* Alteplase in Patients with CVAD Occlusion ^†^ Cumulative Catheter Clearance Rate (%).

First dose	ALF* 0.3 mg	ALF 1 mg	ALF 3 mg	Alteplase 2 mg
	(n = 16)	(n = 16)	(n = 10)	(n = 13)
5 min	13	13	40	0
15 min	13	44	50	0
30 min	25	44	60	23
120 min	38	56	60	46
**Second dose**				
5 min	38	56	60	54
15 min	38	56	60	62
30 min	38	56	60	62
120 min	44	56	80	62

^†^ Data adapted from [29,30]; *ALF = alfimeprase.

Following these favorable results, a Phase III trial was then initiated called SONOMA-2 (Speedy Opening of Non-functional and Occluded catheters with Mini-dose Alfimeprase). The trial was initiated in September 2006 and was a double blind trial comparing efficacy and safety of 3 mg of alfimeprase *vs.* placebo and was to include 303 patients with CVAD occlusion. The primary endpoint was the restoration of function to occluded catheters in 15 minutes. Unfortunately, it was announced in December 2006 that the trial did not meet the end point of restoration of function of occluded central venous catheters in 15 min. Alfimeprase restored catheter function in 15 min but with a p-value of 0.022, it did not meet the more stringent p-value of <0.00125 required of this trial. Also, the trial did not meet established secondary endpoints and enrollment in the new SONOMA-3 trial was temporarily suspended in December 2006. However, SONOMA-3 was reopened in August 2007 as an open-label, single-arm trial of alfimeprase alone using a 10 mg dose at 5 mg/mL in up to 100 patients. The primary endpoint was safety; efficacy was also evaluated. Alfimeprase restored catheter function in ~50% of patients in 15 min and ~60% at 1 h, better than in the SOMOMA-2 trial, but not good enough. By comparison CathfloTM Activase® (alteplase or rt-PA), cleared ~80% of occluded catheters in patients by 2–4 h. Alfimeprase was to restore catheter function with similar efficacy to CathfloTM Activase®, but in a shorter time. In combination with poor results in NAPA-3, Nuvello decided to abandon the development of alfimeprase in March 2008 (http://www.bizjournals.com/sanjose/stories/2008/03/17/daily14.html?t=printable).

Prior to dropping the alfimeprase project, Nuvello had initiated a Phase II clinical trial in acute ischemic stroke (http://www.fiercebiotech.com/node/13455/print). Fast track designation for alfimeprase was granted for this proof of concept study. Stroke is the third leading cause of death in the United States, with about 700,000 cases per year, and a significant cause of long-term disability. Stroke is caused when a blood vessel that is carrying nutrients and oxygen to the brain becomes blocked by a blood clot (ischemic stroke) or ruptures due to some causative factor (hemorrhagic stroke). There is an urgent need for agents that can remove the thrombus in ischemic stroke in patients who present at greater than 3 hours after the event. The acute ischemic stroke trial with alfimeprase was called CARNEROS-1 (Catheter directed Alfimeprase for Restoration of NEurologic function and Rapid Opening of arteries in Stroke). The study was initiated in June 2007 and the first patient was treated in December 2007. The trial was a multi-center, open label, dose escalation study starting with doses of 1, 5 and 10 mg of alfimeprase in ~100 patients with acute ischemic stroke. Patients were treated within 3–9 h of onset of stroke. The endpoints to be observed were safety and efficacy in stroke patients treated with intra-arterial, catheter directed bolus alfimeprase. The primary efficacy endpoint was recanalization of main occlusive lesion within 120 min of alfimeprase treatment; safety was assessed by a lack of symptomatic intracerebral hemorrhage within 24 h of drug administration. Due to lack of enrollment in this trial as well as the failed Phase III trials in CVAD and PAO, Nuvelo decided to terminate this study. Thus, in March 2008, Nuvelo discontinued the clinical development of alfimeprase and the program was shut down [23].

## 7. Conclusions

The results of the Phase I trials to evaluate the safety profile, pharmacokinetics, and thrombolytic activity of escalating doses of alfimeprase in patients with chronic peripheral arterial occlusion (PAO) were very promising and there was limited toxicity [25]. In the Phase II trials (safety and efficacy) there were a low number of major hemorrhagic events in both PAO [18] and CVAD occlusion [18,30] trials. Importantly, there was a lack of intracerebral hemorrhage in all trials. The use of alfimeprase resulted in rapid restoration of arterial patency in <4 h in most PAO patients. In the CVAD occlusion trial, alfimeprase restored patency in <30 min with no evidence of hypotension. In both PAO and CVAD occlusion trials there was no evidence of anti-alfimeprase antibodies. These trials indicated that alfimeprase has the potential to be a potent direct-acting fibrinolytic agent with an excellent safety profile. As observed for fibrolase, alfimeprase is rapidly inactivated by α2M after forming a 1:1 complex; a covalent bond forms between α2M and alfimeprase [22,31]. Inhibition of alfimeprase by α2M in the circulation limits fibrinolytic action to the site of the clot. Extensive studies with human blood serum from several hundred volunteers were used to accurately estimate the level of α2M in human blood; this established an upper limit for alfimeprase use in humans. This analysis revealed that the alfimeprase binding capacity is directly proportional to the measured α2M content. When these data were used to calculate a theoretical dose of alfimeprase on a “mg/kg” basis, the mean estimate for the population was 1.7 mg/kg [8]. Although some individuals were theoretically capable of tolerating nearly 4 mg/kg of alfimeprase, some in the population might tolerate dosages of only 0.6 mg/kg. In view of these results, clinical dosages in the first human trial of alfimeprase did not exceed 0.5 mg/kg. 

Despite these findings, transient bouts of hypotension were experienced at the highest doses in the PAO trial [18,22]. These cases spontaneously resolved or could be easily managed by supportive care including bradykinin receptor antagonist or nitric oxide synthetase inhibitor [24]. Bolus delivery of alfimeprase through an intrathrombus side-hole catheter in the PAO trial represents a mechanical manipulation, which creates channels resulting in clot disruption and run-off of alfimeprase; it is then inactivated in the circulation by α2M. The inability to maintain alfimeprase in the thrombus long enough to lyse the full-length of the clot is probably related to the delivery modality. It appears that there was also a problem with the dose and dosing schedule in addition to the delivery problem. The use of a catheter that sprays alfimeprase into the thrombus requires that the catheter penetrate into the clot and deliver the lytic agent into the depths of the thrombus mass. The danger is that the disrupted clot could dislodge and move distally, creating an embolus most likely due to the mechanical effect of the pulsatile delivery.

Finally, the failures to meet endpoints in the Phase III CVAD occlusion trial [23] and the Phase III PAO trial [26] were problematic as was poor enrollment in the Phase II CARNEROS-1 (stroke) trial (http://clinicaltrials.gov/ct2/show/NCT00499902). Thus, in March 2008 Nuvelo discontinued development of alfimeprase (http://www.fiercebiotech.com/node/20663/print). Is there anything that can be done to salvage this technology in the future? Among the areas that might be considered: the use of adjuvant antiplatelet therapy; adjusting the dosage regimen; drug interactions such as with antiplatelet agents, which may be synergistic in nature; the use of a placebo control for the PAO trials based on the report by Han *et al*. [26]; the use of magnitude of surgical intervention as opposed to avoidance of open vascular surgery as end point for the PAO trial; and finally, further evaluation is warranted to optimize the mode of delivery and dose for alfimeprase. This final issue is critical to the success of alfimeprase and the ability to maximize retention of the drug at the thrombus site and increase lytic activity. 
